# Work addiction risk, stress and well-being at work: testing the mediating role of sleep quality

**DOI:** 10.3389/fpubh.2024.1352646

**Published:** 2024-06-20

**Authors:** Morteza Charkhabi, Abbas Firoozabadi, Laura Seidel, Mojtaba Habibi Asgarabad, Francesco De Paola, Frederic Dutheil

**Affiliations:** ^1^Department of Educational Programs, HSE University, Moscow, Russia; ^2^Department of Psychology, Faculty of Education Sciences and Psychology, Ferdowsi University of Mashhad, Mashhad, Iran; ^3^School of Psychology, University of Ottawa, Ottawa, ON, Canada; ^4^Department of Psychology, Norwegian University of Science and Technology, Trondheim, Norway; ^5^Private Psychotherapy Clinic, Milan, Italy; ^6^Physiological and Psychosocial Stress, UMR CNRS 6024 LaPSCo, Université Clermont Auvergne, Clermont-Ferrand, France; ^7^Preventive and Occupational Medicine, University Hospital of Clermont-Ferrand, Clermont-Ferrand, France; ^8^Wittyfit, Paris, France

**Keywords:** work addiction risk, sleep quality, stress at work, stress at home, mediation

## Abstract

**Introduction:**

Attention to work addiction risk is growing; however, more studies are needed to explore the possible impact of work addiction risk on various aspects of employees’ work and life domains. Although several studies have considered the antecedents or consequences of work addiction risk, this study particularly focuses on sleep quality as a potential explanatory underlying mechanism in the relation between work addition risk and three outcome variables including stress at home, stress at work and well-being.

**Method:**

The data was collected using an online platform and participants consisted of 188 French employees who were selected using simple random sampling method. Participants responded to the survey including the Work Addiction Risk Test (WART), stress at work, well-being, and sleep quality. The data was analyzed using JASP and SPSS-26 programs.

**Results:**

The results revealed that there are significant positive relationships between work addiction risk and both stress at home and at work and negative relationships between work addiction risk and both sleep quality and well-being. In addition, the analyses of the mediation paths suggest the significant mediation role of sleep quality for the link between work addition risk and stress at work as well as the link between work addiction risk and well-being.

**Discussion:**

Given the verified mediating role of sleep quality in the relationship between work addiction, stress and wellbeing, it is recommended that organizations and companies pay particular attention to their employees’ sleep quality.

## Introduction

Nowadays the majority of individuals need to work to cover their basic needs. This has changed the traditional style of families where only men were recognized as the breadwinner of families (1). Currently it is broadly common to observe a family where both spouses work, and they are paid for their inputs in the workplace. One-step beyond this, is that working has changed its meaning from a solo financial source provider to a place or position where an individual can attend, learn, grow and influence an output. These attractive aspects of workplaces have created a competitive climate within workplaces in which individuals tend to invest more inputs in terms of time and energy in their jobs than those required or expected to continue their job or to receive job promotions in their workplace. This phenomenon has been conceptualized as work addiction [(2), Chapter 9].

Work addiction known as work addiction risk (3, 4) is recognized as a public health concern (5, 6) that is accompanied by detrimental consequences for individuals who experience it [e.g., (7)]. Work addiction risk is a worldwide phenomenon that occurs when there is an unconstructive way of involvement with work (5, 6). Workaholic individuals show the tendency to ignore the experience of fatigue and to work hard for a long period until physical complaints appear and stop them from working effectively (8). It is based on the notion that workaholics perform a kind of self-neglect to their life to meet their working standards (9). Although the workaholic employees have the opportunity to choose between working enough and working hard, the majority choose the second option.

There are studies suggesting that work addiction can disrupt the balance between work and family domains, resulting in work–family conflicts (10–12). This occurs when an individual tries to meet the demands of both work and life domains, but a lack of sufficient resources may prevent him from achieving this goal, leading to work–family conflict [e.g., (13)]. According to the Job Demand-Control Theory (14), individuals with a high risk of work addiction are likely to set higher work standards for themselves in response to job uncertainty, competitiveness, economic insecurity, and in attempting to meet these standards, they may experience greater work stress (4, 15). These self-imposed standards may cause individuals to invest considerable time, energy, and effort into their work, leaving them with reduced energy and diminished emotional resources to cope/deal with their life demands (16, 17), leading to an increased feeling of stress at home as well.

Although studies suggest that the work addiction leads to favorable outcomes such as increased job performance (18) or improved career satisfaction (19), there have been a large body of research evidence suggesting that work addiction leads to unfavorable outcomes. For examples, research has shown that work addiction is associated with lower physical health (20), lower mental health (21), increased work–family conflicts (20), lower life satisfaction (22), higher depression rate (4), and higher risk of cardiovascular diseases and stroke (23).

Although majority of the current studies focused on the antecedents or the consequences of work addiction [e.g., (18, 24)], we first focus on the particular link between work addiction risk and the health status of employees. We will then focus on the mediating role of sleep quality as a potential mediator that may explain the influence of work addiction risk on health-related indicators. Sleep quality is defined as an individual’s self-satisfaction with all aspects of the sleep experience (25).

**Figure 1:**
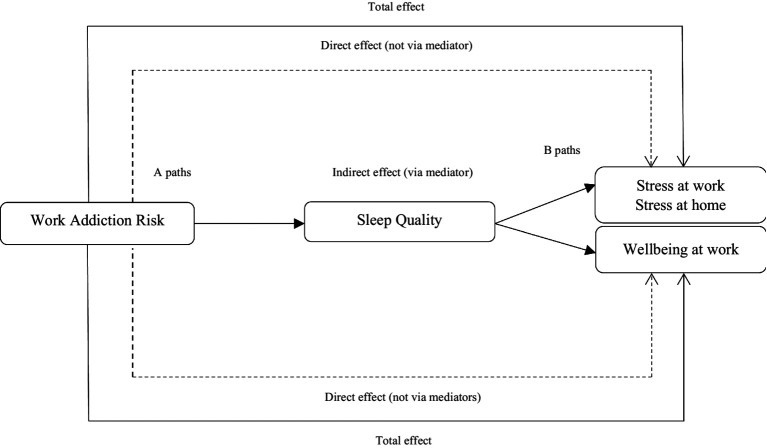
A proposed model of the direct and indirect paths.

Figure 1 illustrates a conceptual model of the relationships between the research variables. In this model, work addiction risk is considered as the predictor, sleep quality as the mediator and stress and well-being as the outcome variables.

### A review on the work addiction risk and health-related outcomes

One of the models that can help to understand work addiction is Latent Deprivation Model (26). According to this model, besides the manifest function of earning money, work contains several latent functions that guarantee the satisfaction of other psychological needs of humans such as sense of purposefulness and being active, relatedness to others, and self-determination. Having access to the latent functions of work is essential for sustaining employees’ mental health and well-being. Due to the increasing inter-organizational competitions and in order to reach the expected perspectives, organizations are driven to design jobs by which they maximize their employees’ sense of involvement with their jobs. Additionally, the changes in the nature of jobs made during the last decades of the 21st century, such as communication technology advances and the development of organizational tools assessing labor productivity and job performance, has furthered entered work into the life of employees (27, 28).

Due to the important meaning that work plays in today’s life as well as experiencing the more demanding nature of work, the employees may spend excessive time and put greater effort on their work-related tasks at work or even at home and be less willing to delegate their work-related activities [e.g., (1, 29)]. The prevalence of this workstyle may bind individuals to their occupations, a phenomenon known as work addiction risk. Work addiction has been described as one of the main addictions of the 21st century (30) that is defined as “the stable tendency of excessive and compulsive working” [(31), p. 1] beyond what is required or expected [(2), Chapter 9]. Work addicted individuals are described as those who spend a considerable amount of time in their work activities, are constantly preoccupied with work-related issues and work more than what is formally expected to meet their work requirements mainly due to an inner compulsion (18, 32). According to Taris et al. (33) and Burke and Cooper (2), the experience of work addiction risk is proposed to include two components. First is the behavioral component that comprises devoting exceptional time to work and working excessively hard (i.e., overwork). Second is the psychological component that comprises thinking obsessively about work and working compulsively.

Work addiction is proposed to share similarities with other behavioral addictions in terms of compulsive involvement with specific activities that might be perceived rewarding by employees as they serve to diminish the force of certain obsessions (31, 34). Moreover, unlike other behavioral addictions, employees who are addicted to work may engage in activities that are socially acceptable and even encouraged by their employers and organizations. Due to expending higher effort to attain work-related goals, employees are more likely to experience a sense of achievement and higher levels of job performance and satisfaction (18, 35). Work addiction might also act as a coping strategy that alleviates the degree of negative emotions that individuals experience in their personal life domain. Therefore, it can be associated with less preoccupation of employees with other areas of their lives (36).

In spite of the mentioned positive aspects, work addiction, as an excessive pattern of involvement and similar to other behavioral addictions, can be detrimental to the employees’ health and well-being (20, 31, 37). According to World Health Organization (WHO) work stress is defined as ‘the response people may have when presented with work demands and pressures that are not matched to their knowledge and abilities and which challenge their ability to cope (38). Given the compulsion aspect, work addiction can be associated with higher levels of job demands and workload (39, 40). In their research, Dutheil et al. (4) showed a five times more risk of work addiction among employees with high job demands compared to those with low job demands. Experiencing higher pressure of work under the demanding circumstances can be associated with higher levels of stress that people may experience during working time (41, 42). Dealing with high levels of workload and working for a longer period of time require higher expenditure of effort during working time that in turn will lead to psychological exhaustion (43) and impaired well-being (44) through the depletion of employees’ energy resources (45). Moreover, workaholics are more likely to being mentally engaged with work-related issues even during their non-work time (46). Continued exposure to work demands during off-job time leads to the prolonged psychological activation spilling over from the work to the home domain that is detrimental for well-being over time. Research has shown that working under high workload condition is associated with high work-related ruminative thinking (47, 48) that itself will lead to health impairment (49) through impeding the recovery process (50). Work addiction risk may also influence the employees’ psychological well-being through increasing work–family conflict (12, 20, 51, 52). It has been also proposed that workaholics can be poor performers as they are more likely to sacrifice the quality of their actions to successfully accomplish work-related tasks to the quantity of tasks, they can accomplish during a limited period of time at lower levels than required standards (53). Therefore, they may experience lower job performance (39), impaired social functioning and higher interpersonal conflicts at their workplace (54). Employees are then more likely to perceive work as being more stressful and experience negative health consequences.

Well-being is a multifaceted construct with different definitions. However, one of the well-known definitions of wellbeing is presented by World Health Organization (WHO). According to WHO (55) ‘Health is a state of complete physical, mental and social well-being and not merely the absence of disease or infirmity.’ In this study the overall wellbeing was used. Previous research has shown the positive link between work addiction risk and health related outcomes such as exhaustion, depression, life dissatisfaction, and sleep difficulties [e.g., (4, 22, 53, 56, 57)]. Regarding the consequences of work addiction risk on employees’ personal and work lives, the current study particularly examines the relationship between work addiction risk and three health-related outcomes including stress at home, stress at work and general well-being in a sample of French employees. We hypothesize that:

*Hypothesis 1*: work addiction risk is positively associated with (a) stress at work and (b) stress at home of employees.

*Hypothesis 2*: work addiction risk is negatively associated with well-being of employees.

### Mediating role of sleep quality

Workaholics experience higher levels of workload and job strain not only during their working time but also during the time after work and during off-days including weekends and holidays. Research [e.g., (50, 58, 59)] has shown that the extent to which employees can unwind from work related issues and engage in activities that facilitate the process of replenishing depleted resources during their non-work time (i.e., recovery from work) is crucial for employees’ well-being. Addiction to work risk is associated with higher levels of being mentally exposed to work-related issues during non-work time (60–62). Research showed that employees with high levels of work-related ruminative thinking are more likely to experience sleep difficulties compared to employees with low levels (47, 49, 63). In a diary study, Syrek et al. (64) showed that unfinished tasks and work-related thinking are associated with sleep impairment.

Sleep quality has been established as an important factor for sufficient recovery and for preventing the long-term negative consequences of job strain on well-being. The brain needs a good quality of sleep to replenish the energy resources that a person has lost during the day (65). These energy resources are assumed to be depleted more in workaholic employees as the result of their daily overwork (4), experience of work-related thinking (66) and work–family conflicts (12, 52), compared to non-workaholic employees. Therefore, due to an impaired sleep, the depleted resources are not sufficiently replenished and people are more likely to experience negative affect (67), acute and chronic fatigue (63), and exhaustion (68). Once the energy resources are not successfully replenished due to the low quality of sleep (45), individuals’ failure to regulate their unpleasant feelings such as stress and negative affect increases. According to Carver and Scheier (69), regulating affective states is an intense and effortful process that is directly depended on the available level of energy resources. The prolonged activation of unpleasant feelings then further drains resources and has negative consequences on well-being. Using a cross-sectional study, this study aims to shed light on the relation between work addiction risk and three health-related outcomes (i.e., stress at home, stress at work and well-being) by considering the mediation role of sleep quality. We hypothesize that:

*Hypothesis 3:* sleep quality mediates the association between work addiction risk and (a) stress at work and (b) stress at home.

*Hypothesis 4:* sleep quality mediates the association between work addiction risk and well-being.

## Method

### Procedure

Employees were selected through a French online platform known as WittyFit^1^ and used by several French companies (4, 70). WittyFit initially was developed in collaboration with the University Hospital of Clermont-Ferrand in France and is a web-based platform to monitor, measure and enhance employees’ well-being at work. Both National Commission for Data Protection and Liberties (CNIL), and the South-East VI ethics committee (clinicaltrials.gov identifying number NCT02596737) approved the content and instruments of this study. The nature of data collection in this platform is unanimous and there was no connection between researchers and participants. Employees received an e- questionnaire through the WittyFit platform and they were invited to read all research instructions and participate voluntarily in this study.

### Participants

The HR department of those companies that were registered in the WittyFit platform^2^ invited participants and they agreed to participate in this study. 188 out of 1,580 registered French employees in this platform were recruited based on a simple random sampling method. Of those, 47.1% were female and 39% were male. 13.9% of participants did not report their gender. Mean age of participants was 41.83. No intervention was performed, and no exclusion criteria were established to recruit the participants. Considering the nature of data collection, which was based on an online anonymous questionnaire, the ethics committee waived the requirement for participants’ consent.

### Measures

#### The work addiction risk test (WART)

This scale was developed by Robinson et al. (7, 71, 72) from the symptoms reported by clinicians caring for patients with work addiction risk (5, 72). The WART assesses 25 statements on a 4-point Likert scale from never true (1) to always true (4). The total score ranges from 25 to 100 which higher scores reflecting higher work addiction risk. Scores from 25 to 56 are defined as a low-risk of work addiction risk; from 57 to 66 as a medium-risk and from 67 to 100 as a high-risk (7, 73). An item example of this scale is “*I prefer to do most things myself rather than ask for help*.” We previously validated the French version of the WART (74). In this study, the Cronbach’s Alpha was 0.90.

#### Sleep quality

This construct was evaluated using visual analog scales (VAS), i.e., by moving a cursor on a horizontal, non-calibrated line of 10 cm, ranging from very low (0) on the left to very high (10) on the right (75, 76). It was measured by using the following item “*what is the quality of your sleep*.” Higher values represent higher sleep quality.

#### Perceived stress at work

To measure perceived stress at work we used a visual analog scale (VAS), i.e., by moving a cursor on a horizontal, non-calibrated line of 10 cm, ranging from very low (0) on the left to very high (10) on the right (75, 76). It was measured by using the following item “*what is your stress level at work*.” Higher values represent higher stress at work.

#### Perceived stress at home

It was assessed using visual analog scale (VAS), i.e., by moving a cursor on a horizontal, non-calibrated line of 10 cm, ranging from very low (0) on the left to very high (10) on the right (75, 76). It was measured by using the following item “*what is your stress level at home*.” Higher values represent higher stress at home.

#### Well-being

This construct is evaluated using visual analog scale (VAS), i.e., by moving a cursor on a horizontal, non-calibrated line of 10 cm, ranging from very low (0) on the left to very high (10) on the right (75, 76). It was measured by using the following item “*what is your level of wellbeing*.” Higher values represent higher well-being.

#### The socio-demographic characteristics questionnaire

It assessed age, gender, height, weight, occupational group, education level, family situation, work characteristics, quantity of sleep, and physical activity.

### Data analysis

To calculate the required number of participants of this study we used previous studies (77). The sample size also followed the recommendations of G*power statistical program^3^ to meet minimum number of participants for data collection. Statistical analyses were performed using SPSS-26 program and JASP program. Correlation analysis and mediation analysis were used to investigate the direct and indirect association between the research variables.

## Results

### Descriptive statistics

The descriptive statistics of the research instruments (means and standard deviations) and the Pearson correlations between the variables are displayed in Table 1. As the table shows, work addiction risk is negatively associated with sleep quality (*r* = −0.332, *p* < 0.001) and well-being (*r* = −0.375, *p* < 0.001) and is positively associated with stress at work (*r* = 0.413, *p* < 0.001) and stress at home (*r* = 0.400, *p* < 0.001). Sleep quality is negatively associated with stress at work (*r* = −0.312, *p* < 0.001) and stress at home (*r* = −0.188, *p* < 0.001) and is positively associated with well-being (*r* = 0.542, *p* < 0.001). There is also a negative association between both stress at work (*r* = −0.311, *p* < 0.001) and stress at home (*r* = −0.384, *p* < 0.001) and well-being.

**Table 1:** Correlation matrix between the research variables in the present study (*n* = 161).

	Variable	*M*	*SD*	1	2	3	4	5	6	7
1	Gender	0.45	0.49	–						
2	Age	41.83	11.85	−0.16	–					
3	Education	4.52	0.92	−0.126	−0.147	–				
4	Work addiction risk	57.82	11.23	−0.250*	0.219**	0.219**	–			
5	Sleep quality	56.51	27.79	0.146	0.022	0.067	−0.332**			
6	Stress at work	56.17	24.77	−0.036	−0.062	0.109	0.413**	−0.312**		
7	Stress at home	35.39	25.23	−0.080	−0.161*	−0.008	0.400**	−0.188**	0.439**	
8	Well-being	62.61	21.85	0.139	0.052	0.110	−0.375**	0.542**	−0.311**	−0.384**

*p < 0.05, **p < 0.01.

**Figure 2:**
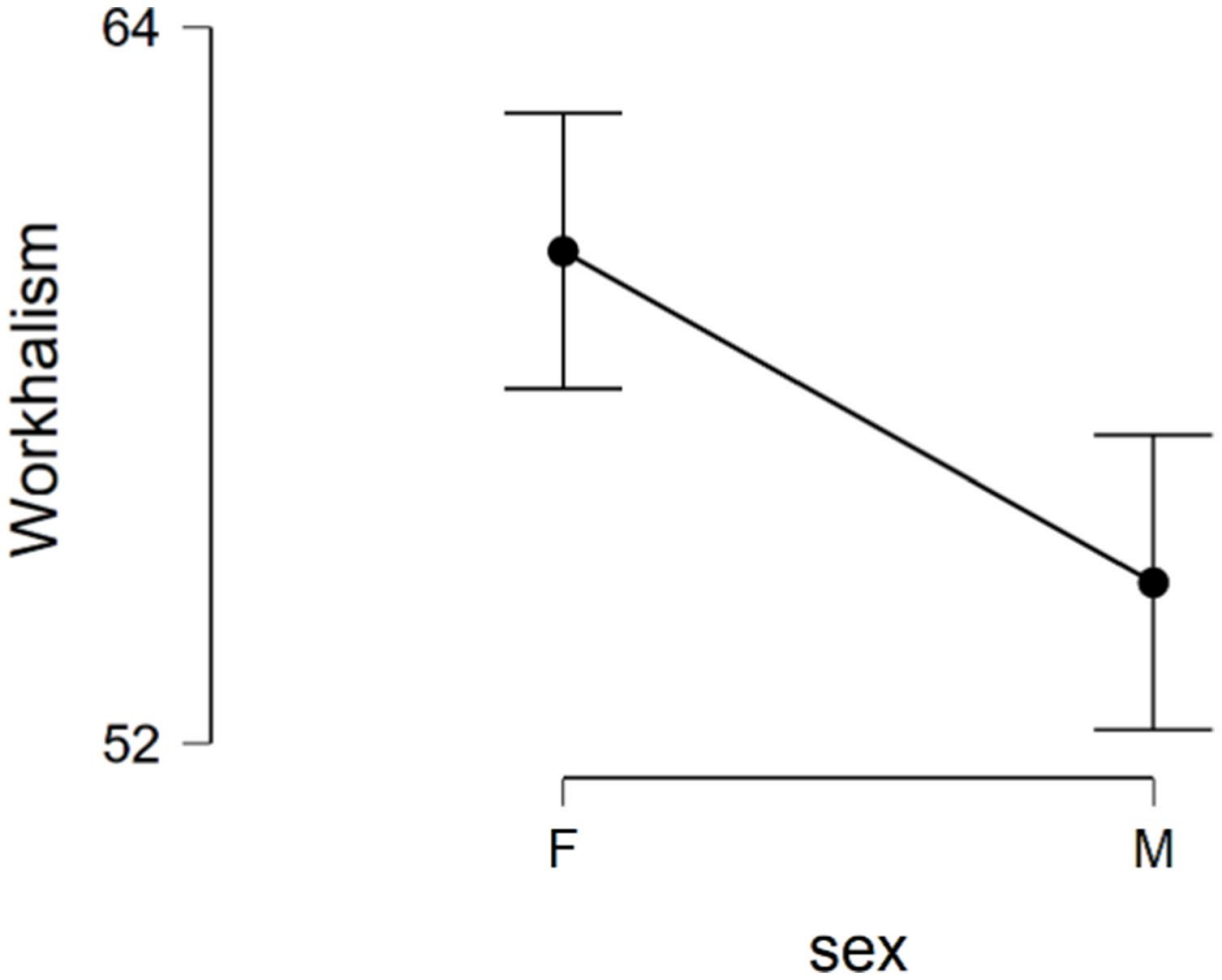
Distribution of reported work addiction risk across gender.

The results of independent sample t-test suggested that there is no difference between male and female participants in predicting, mediator and outcome variables except for work addiction risk. The results are displayed in Table 2. According to this table, female participants reported higher work addiction risk than male participants and this difference was statistically significant (*t* = 3.26, *df* = 159, *p* = 0.001). Figure 2 displays the distribution of reported work addiction risk across gender.

**Table 2:** Results of independent samples T-Test of studied variables between male (*n* = 73) and female (*n* = 88) participants.

						95% CI for mean difference		
	*t*	*df*	*p*	Mean difference	SE difference	Lower	Upper	Cohen’s d
Work addiction risk	3.262	159	0.001	5.551	1.702	2.190	8.912	0.516
Sleep quality	−1.842	156	0.067	−8.120	4.407	−16.825	0.586	−0.295
Stress at work	0.444	156	0.657	1.759	3.960	−6.063	9.581	0.071
Stress at home	1.000	156	0.319	3.956	3.958	−3.862	11.775	0.160
Well-being	−1.750	156	0.082	−6.138	3.508	−13.067	0.792	−0.280

Student’s *t*-test.

### Testing the mediating role of sleep quality

To test the mediating hypotheses, a regression model according to Figure 1 was constructed. We used JASP statistical program version 0.14.1.0^4^ to construct this model. To test the mediation paths, we installed the SEM package on this program. The results are displayed in Table 3. As the table shows, work addiction risk positively predicted stress at work (*β* = 0.031, *p* < 0.01). In addition, sleep quality statistically mediated the indirect association between work addiction risk and stress at work (*β* = 0.037, *p* < 0.001). The results also suggested that the total effect of work addiction risk on stress at work is statistically significant (*β* = 0.006, *p* < 0.01). According to the table, work addiction risk also positively predicted stress at home (*β* = 0.034, *p* < 0.001). Although the total effect of work addiction risk on stress at home was significant (*β* = 0.036, *p* < 0.001), the sleep quality did not mediate the association between work addiction risk and stress at home (*β* = 0.002, *p* = 0.393).

**Table 3:** Results of mediating role of sleep quality between work addiction risk and stress and well-being (*n* = 161).

					95% Confidence interval	
Effect	*β*	*SE*	*z-value*	*p*	*LLCI*	*ULCI*
Direct effect of work addiction risk on stress at work	0.031	0.0006	4.981	0.001	0.019	0.044
Indirect effect of number of work addiction risk on stress at work via sleep quality	0.006	0.002	2.421	0.015	0.001	0.011
Total effect of work addiction risk on stress at work	0.037	0.0006	6.140	0.001	0.025	0.049
Direct effect of work addiction risk on stress at home	0.034	0.0006	5.293	0.001	0.021	0.047
Indirect effect of number of work addiction risk on stress at home via sleep quality	0.002	0.002	0.854	0.393	−0.002	0.006
Total effect of work addiction risk on stress at home	0.036	0.006	5.905	0.001	0.024	0.048
Direct effect of work addiction risk on wellbeing	−0.020	0.0006	−3.425	0.001	−0.031	−0.008
Indirect effect of number of work addiction risk on wellbeing via sleep quality	−0.014	0.004	−3.997	0.001	−0.021	−0.007
Total effect of work addiction risk on wellbeing	−0.034	0.006	−5.465	0.001	−0.046	−0.022

Delta method standard errors, normal theory confidence intervals, ML estimator. *p < 0.05, **p < 0.01.

The results in Table 3 also suggest that the work addiction risk negatively predicts well-being (*β* = −0.020, *p* < 0.001). The indirect path from work addiction risk to well-being through sleep quality was statistically significant (*β* = −0.014, *p* < 0.001), which means that the sleep quality acts as the mediator of the association between work addiction risk and well-being. Moreover, the total effect of the work addiction risk on well-being was found statistically significant (*β* = −0.034, *p* < 0.001).

**Figure 3:**
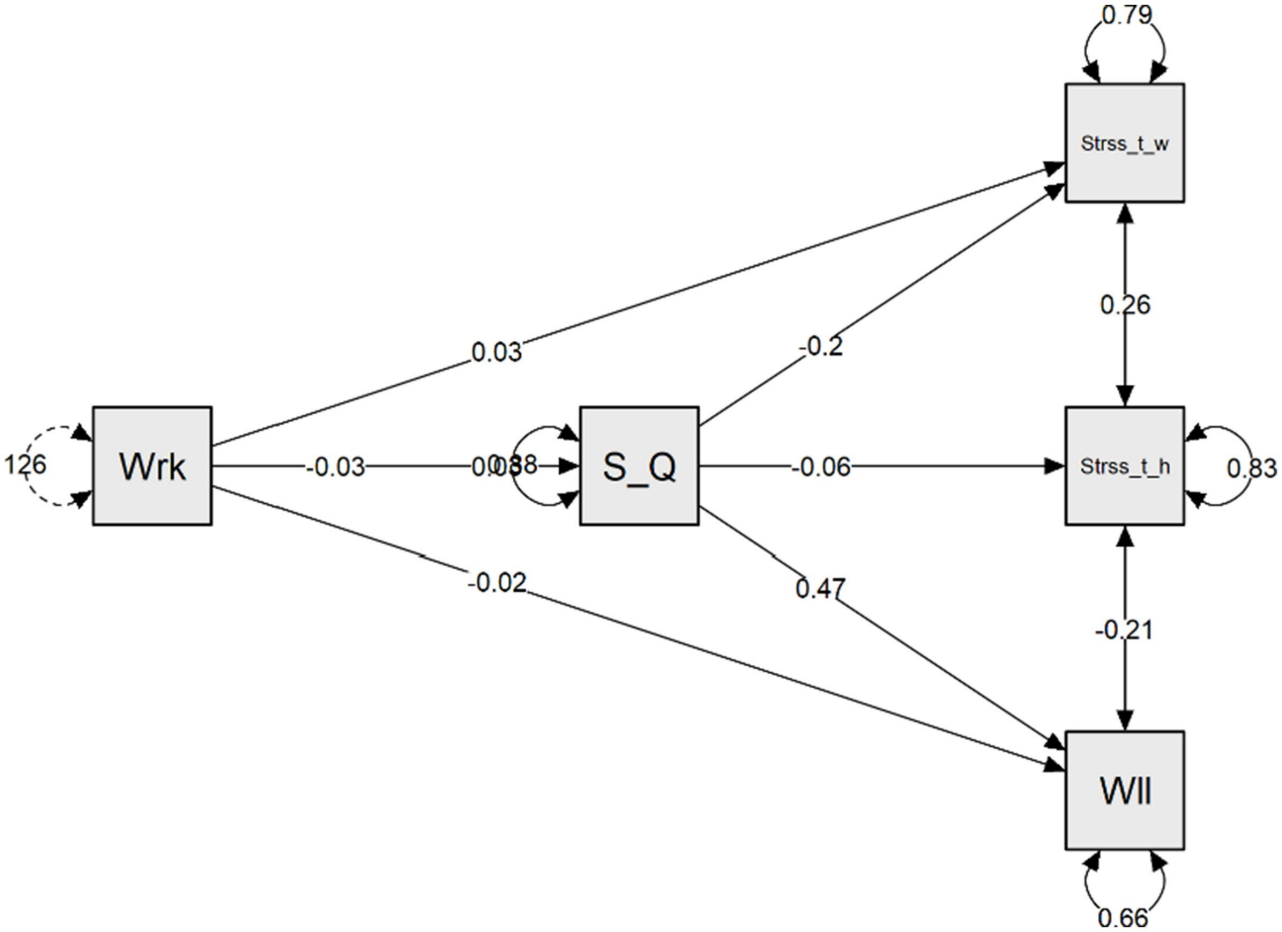
A mediation model of the relations between research variables. Wrk, work addiction risk; S_Q, sleep quality; Stress_t_w, Stress at work; Stress_t_w, Stress at home; Wll, wellbeing.

Figure 3 displays the tested mediation model related to the direct and indirect paths between work addiction risk, stress and wellbeing.

**Figure 4:**
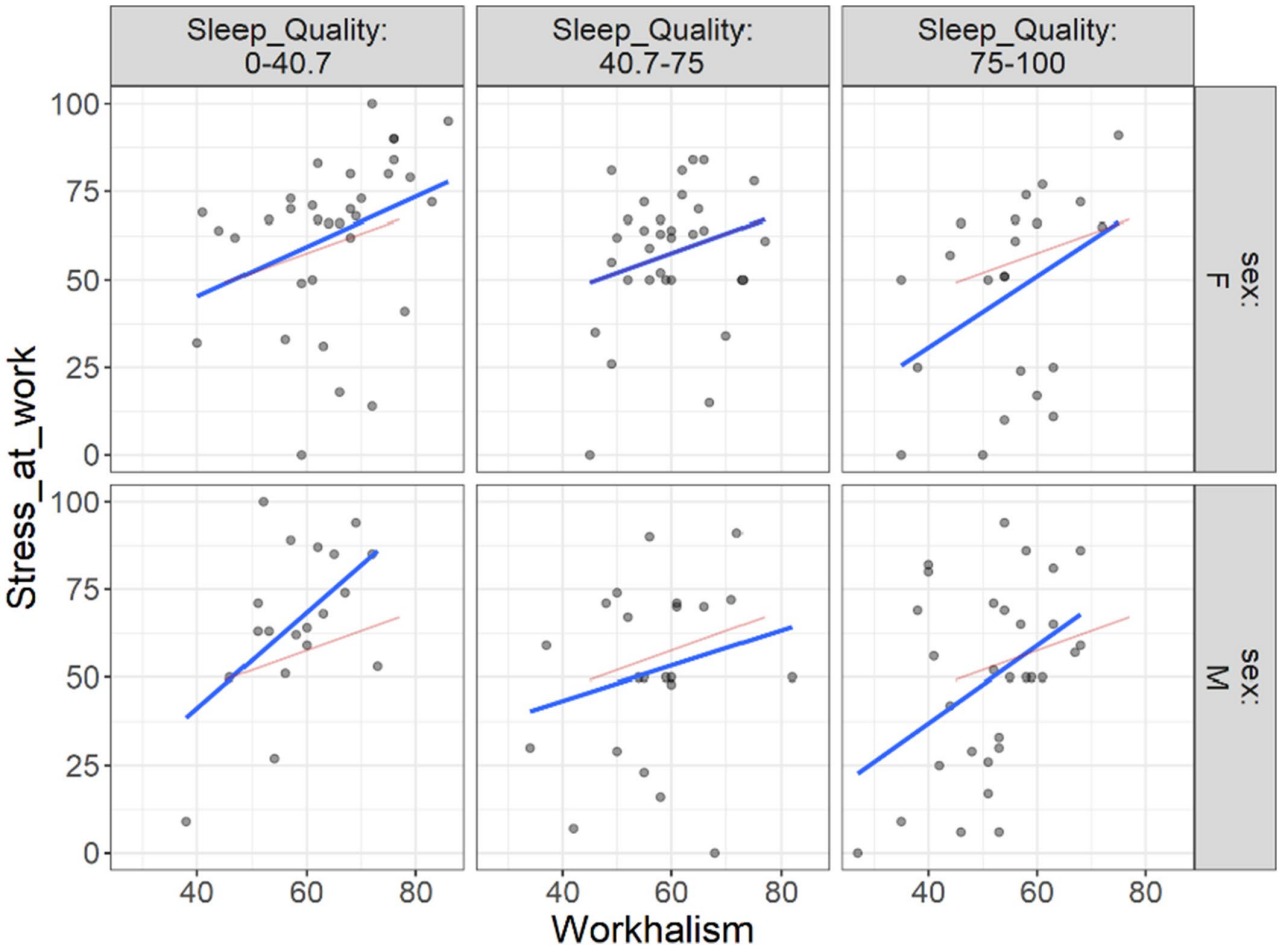
Flexplot of the relations between work addiction risk and stress at work at different levels of sleep quality across gender.

Additionally, we attempted to demonstrate the extent to which the link between work addiction risk and various outcomes in different levels of sleep quality may vary across male and female individuals. This was performed due to the different work addiction rate we found in Table 2. To display this, we drew flexplots that demonstrate these situations. Figure 4 shows by increasing the work addiction risk, the stress at work also is increased. This increase, when sleep quality is low is higher than when the sleep quality is at highest level. Furthermore, this increase in lower level of sleep quality is seen higher among males than females.

**Figure 5:**
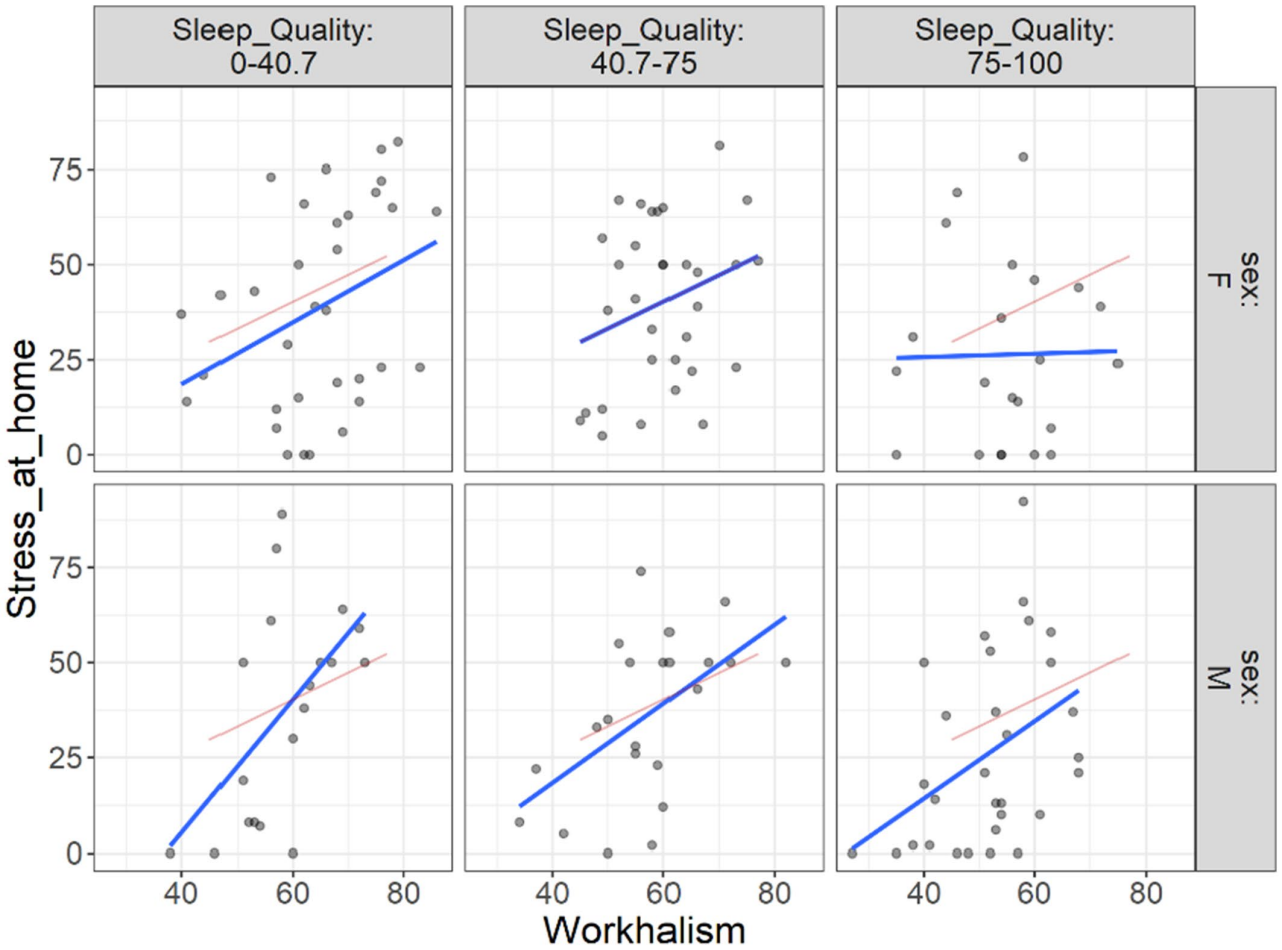
Flexplot of the relations between work addiction risk and stress at home at different levels of sleep quality across gender.

Figure 5 shows a situation in which the link between work addiction risk and stress at home is more strongly influenced by the level of sleep quality in male and female participants. In doing so, this link is perceived more positively and higher for male than for female when the sleep quality of both is low but when the sleep quality is reported high the link still is strong for male and almost much lower for female participants.

**Figure 6:**
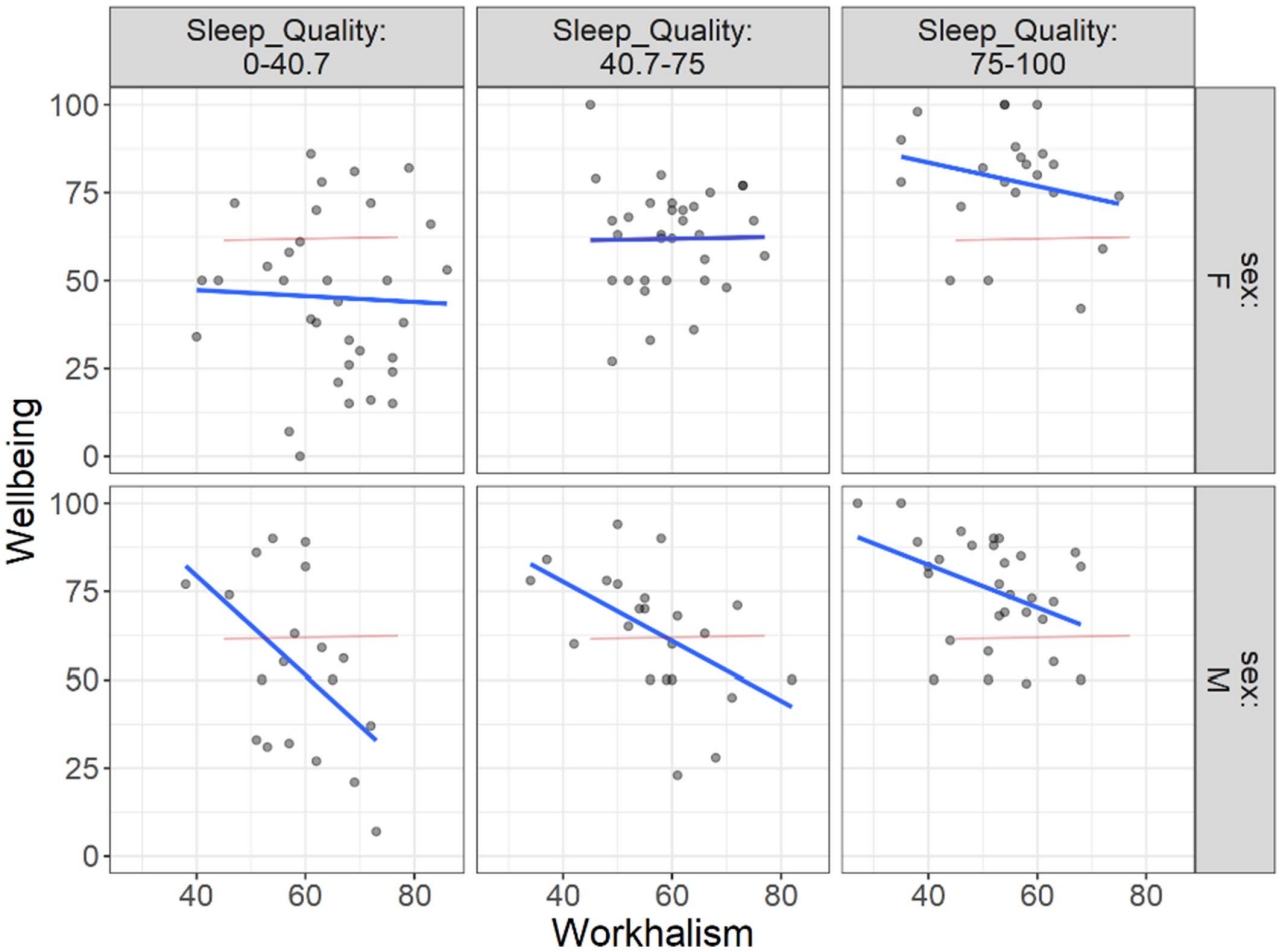
Flexplot of the relations between work addiction risk and well-being at different levels of sleep quality across gender.

As Figure 6 illustrates by increasing the work addiction risk, the well-being is reduced. This reduction is more tangible when sleep quality is reported at lowest level in males than in females. However, this link seems to be similar between male and females when the sleep quality is reported at its highest level. Figure 6 demonstrates this result.

## Discussion

The objective of this study was to reveal the type and strength of the relation between work addiction risk and health-related outcomes. Our primary point of attention was to test the influence of work addiction risk on well-being as well as on stress at home and work. In this regard, the findings supported and replicated some of the previous studies. More specifically, work addiction risk is negatively associated with well-being and positively is associated with both type of stress. This is consistent with previous studies indicating that employees that have higher work addiction risk also report greater stress at work and poorer well-being [e.g., (4, 20, 21)]. It should be noted that although work addition risk is significantly linked with both well-being and stress at work, hence the link between work addiction risk and stress at work is stronger.

As we considered sleep quality as the mediator of the link between work addiction risk and stress and well-being, thus we needed to check the Baron and Kenny (78) mediation condition before we ensure that it can be the mediator. In doing so, we found a negative association between work addiction risk and sleep quality. It is in line with previous studies that found individuals high on work addiction demonstrated significantly higher levels of daytime sleep dysfunction than individuals who rated low on work addiction (79). Sleep quality itself was also positively associated with well-being and negatively associated with stress at home and work. The results are consistent with previous studies that higher level of sleep quality is associated with higher level of well-being [e.g., (80, 81)] and higher level of sleep quality is associated with lower job stress (82, 83) and experience of insomnia (84). The link between sleep quality and well-being was found to be stronger than the link between sleep quality and stress at work, suggesting that if an employee’s sleep quality is negatively impacted, we may expect to observe its proximal symptoms such as decreased well-being at first and vice versa.

According to mediating findings, it is suggested that the work addiction risk is more likely to influence well-being and stress at work through reduced sleep quality. However, sleep quality appeared to be a stronger mediator of work addiction risk and well-being. In addition, according to mediation paths, work addiction risk appears to be the stronger predictor of stress at work and sleep quality appears to be the stronger predictor of well-being. Thus, when there is a likelihood of work addiction risk, individuals with lower sleep quality are likely to experience well-being –related problems and demonstrate it in the form of stress at work. An individual with sleep dysfunction may not be able to do his/her job duties and may put her in a situation to feel stressed. In a rather similar cross-sectional study conducted by Spagnoli et al. (85) in Italy, they tested a mediation model of work addiction risk where sleep quality was set as the mediator of the link between work addition risk and daytime sleep. They found that sleep quality mediated the relationship between work addiction and daytime sleepiness. In another study, Cheng et al. (86) found that not workaholic’s individuals but nurses with high work–family conflicts due to lower sleep quality experienced lower self-perceived heath. In this study sleep quality was the mediator of the link between work–family conflict and health status. An explanation for this finding could be that addicted employees to work have to sleep late and less than non-addicted employees do. The change in the quality of sleep may change the normal concentration level and consciousness at work resulting in not being able to finish their working tasks or increase the fear that their working duties may not be completed during working hours.

## Suggestions and limitations

This study carries some limitations. First, we did not investigate the role of all demographic variables (i.e., education level, marriage status, work experience, type of work contract, etc.) in the extent to which work addiction can be perceived or can lead to health-related outcomes. Although it could be an interesting research idea to examine, it was out of the scope of this study. We suggest future studies take this gap into consideration. Second, we used self-administer scales to collect data. In doing so, we did not have a full control on the social desirability bias of participant that may influence their responses. Third, we collected data using a cross-sectional design in which we did not track our findings over time. We suggest future studies to use other research designs such as longitudinal designs to check the strength of the findings over time.

The results can be used to encourage employees to move toward enhancing the quality of their sleep as an efficient way to cope with occupational stressors. Moreover, employees at risk of work addiction can monitor and receive additional resources and support, such as counseling services or stress management educational workshops, to better manage work addiction risk. The findings also suggest workplaces offer flexible work plans that can promote a healthier work-life balance among employees. Also, workplaces may develop and conduct workshops and training classes to educate employees about prevention strategies against work addiction risk.

## Conclusion

This study one step further completed what the previous studies have found on the detrimental impact of work addiction risk on well-being status of employees through highlighting the specific role of sleep quality of employees on the extent to which the strength of work addiction risk influence stress level and well-being of employees. This draws the attention of workplace managers to the way employees may develop and experience the negative outcomes of work addiction risk. This could be particularly important for those organizations that try to reduce or cut their expenses in terms of employees’ insurances as well as absenteeism and presentism of employees. Moreover, researchers may use these findings to develop or implement interventions that can either reduce work addiction risk or enhance the sleep quality of employees. In addition, employees at risk of work addiction can be identified, monitored, and supported by interventional programs such as counseling services. Besides, prevention strategies to work addiction risk can also be viewed as an effective way and be examined by scientists to measure the extent to which they can contribute to reduction of work addiction risk among employees.

## Author’s Note

The abstract of this article was presented at the European Congress of Psychology (ECP), Slovenia, Ljubljana, as an oral presentation with interim findings. The oral’s abstract was published in a special issue of *Horizons of Psychology*, 31, 59–447 (2022).

## Data availability statement

The raw data supporting the conclusions of this article will be made available by the authors, without undue reservation.

## Ethics statement

The studies involving humans were approved by National Commission for Data Protection and Liberties (CNIL), and the South-East VI ethics committee (clinicaltrials.gov identifying number NCT02596737). The studies were conducted in accordance with the local legislation and institutional requirements. The participants provided their written informed consent to participate in this study.

## Author contributions

MC: Writing – review & editing, Writing – original draft, Software, Resources, Formal analysis, Data curation. AF: Writing – review & editing, Writing – original draft, Visualization, Validation, Supervision, Resources, Methodology. LS: Writing – review & editing, Visualization, Validation, Resources, Methodology, Investigation. MH: Writing – review & editing, Visualization, Validation, Supervision, Software, Methodology, Investigation. FP: Writing – review & editing, Visualization, Validation, Supervision, Investigation, Conceptualization. FD: Writing – review & editing, Writing – original draft, Validation, Supervision, Resources, Methodology, Investigation, Funding acquisition, Data curation, Conceptualization.

## Footnotes

^1^https://wittyfit.com/

^2^https://wittyfit.com/en/

^3^https://www.psychologie.hhu.de/arbeitsgruppen/allgemeine-psychologie-und-arbeitspsychologie/gpower

^4^https://jasp-stats.org/
